# Supplementary material for: Measuring reading time: Comparing logged and self-reported data in relation to reading skills
Source: PLoS One. 2026 Apr 17;21(4):e0344853. doi: 10.1371/journal.pone.0344853 (PMC13089729; doi:10.1371/journal.pone.0344853)
Supplement: Attachment — Submitted filename: Review Plos One.docx [file pone.0344853.s001.docx]

First, I would like to congratulate the research team on their work, as it represents a serious and methodologically sound proposal that brings an innovative perspective to the field of reading research. These comments should be understood as an external viewpoint offering suggestions to be considered in the context of a solid piece of work, rather than as negative criticism.

**Required Revisions and Recommended Changes**

The manuscript addresses a relevant methodological question and is generally well executed; however, several concrete revisions are required to improve clarity, balance, and methodological transparency.

First, the **Introduction should be refocused**. Sections describing biases in self-reported measures and diary methods are repeated across paragraphs. These should be condensed, with redundant explanations removed, so that the introduction more clearly leads to the study’s main contribution. The Introduction is relatively brief compared to the rest of the manuscript. Considerable emphasis is placed on reading time, but the manuscript does not sufficiently substantiate why reading time is so important. What positive outcomes does it influence? Reading competence? Why are the European Recommendations on Key Competences (which explicitly address reading competence), or frameworks such as those from the OEI or PIRLS, not mentioned? Likewise, why is there no reference to other existing applications, such as TECLEED? It would be valuable to state explicitly that reading time has a positive impact on reading competence, which in turn positively affects the overall teaching–learning process. Greater reading practice is associated with fewer reading difficulties (see, for example, <https://doi.org/10.1371/journal.pone.0295606> and <https://doi.org/10.24310/isl.20.2.2025.20620>).

Second, the **rationale for key methodological decisions must be made explicit**. The authors should clearly justify (a) the choice of a 14-day data collection period, (b) the exclusion of reading sessions shorter than one minute, and (c) the use of winsorization for extreme values. Each decision should be briefly supported by references, prior literature, or sensitivity considerations to improve reproducibility.

Third, **statistical reporting should be strengthened**. Effect sizes should be systematically reported alongside p-values for all main comparisons and regression models. In addition, the handling of missing data through grade-level mean imputation requires further justification, as this method may reduce variance and affect correlations with reading fluency. A short discussion of its potential impact should be added.

Fourth, the **Results section should be streamlined**. Detailed numerical descriptions that do not directly address the research questions—particularly repeated confirmations of non-significant grade-level differences—should be shortened or moved to supplementary materials to improve readability and focus.

Fifth, the **Discussion needs to be more balanced**. While the advantages of the logged measure are convincingly presented, the limitations of app-based data collection should be stated more explicitly. These include parental compliance, the possibility of unrecorded independent reading, age-related supervision differences, and potential reactivity effects. Addressing these limitations would strengthen the credibility of the conclusions.

Sixth, the **implications section should be expanded**. The authors should clarify how the findings apply to large-scale, longitudinal, or cross-cultural studies, and discuss practical constraints related to implementing app-based logging in broader samples.

Finally, **minor editorial revisions are required**. Several sentences are overly long and should be simplified, repeated definitions (e.g., of the logged measure) should be reduced, and clearer subheadings in the Discussion would improve structure and reader guidance.

Overall, the manuscript is strong, but I think these specific revisions are necessary to enhance clarity, methodological rigor, and overall impact.
